# Digital Health Solutions and State of Interoperability: Landscape Analysis of Sierra Leone

**DOI:** 10.2196/29930

**Published:** 2022-06-10

**Authors:** Emeka Chukwu, Lalit Garg, Edward Foday, Abdul Konomanyi, Royston Wright, Francis Smart

**Affiliations:** 1 Department of Computer Information Systems Faculty of Information and Communication Technology University of Malta Msida Malta; 2 Directorate of Planning, Policy, and Information Ministry of Health and Sanitation Freetown Sierra Leone; 3 Directorate of eGovernment Ministry of Information and Communication Freetown Sierra Leone; 4 Monitoring and Evaluation Unit Health and Nutrition UNICEF Freetown Sierra Leone

**Keywords:** digital health, mHealth, mobile health, eHealth, Health information and communication technologies, Sierra Leone, big data, HIE, interoperability

## Abstract

**Background:**

The government and partners have invested heavily in the health information system (HIS) for service delivery, surveillance, reporting, and monitoring. Sierra Leone’s government launched its first digital health strategy in 2018. In 2019, a broader national innovation and digital strategy was launched. The health pillar direction will use big data and artificial intelligence (AI) to improve health care in general and maternal and child health in particular. Understanding the number, distribution, and interoperability of digital health solutions is crucial for successful implementation strategies.

**Objective:**

This paper presents the state of digital health solutions in Sierra Leone and how these solutions currently interoperate. This study further presents opportunities for big data and AI applications.

**Methods:**

All the district health management teams, all digital health implementing organizations, and a stratified sample of 72 (out of 1284) health facilities were purposefully selected from all health districts and surveyed.

**Results:**

The National Health Management Information System’s (NHMIS’s) aggregate reporting solution populated by health facility forms HF1 to HF9 was, by far, the most used tool. A health facility–based weekly aggregate electronic integrated disease surveillance and response solution was also widely used. Half of the health facilities had more than 2 digital health solutions in use. The different digital health software solutions do not share data among one another, though aggregate reporting data were sent as necessary. None of the respondents use any of the health care registries for patient, provider, health facility, or terminology identification.

**Conclusions:**

Many digital health solutions are currently used at health facilities in Sierra Leone. The government can leverage current investment in HIS from surveillance and reporting for using big data and AI for care. The vision of using big data for health care is achievable if stakeholders prioritize individualized and longitudinal patient data exchange using agreed use cases from national strategies. This study has shown evidence of distribution, types, and scale of digital health solutions in health facilities and opportunities for leveraging big data to fill critical gaps necessary to achieve the national digital health vision.

## Introduction

### Background

The Ministry of Health and Sanitation (MoHS) inaugurated an eHealth coordination hub in 2017 to facilitate the systematic application of digital health solutions (or services and applications) for health systems improvement through data [1]. This culminated in the launch of the first national digital health strategy for 2018-2023 [2]. In 2019, the Directorate of Science Technology and Innovation at the Presidency also launched a broader National Innovation and Digital Strategy for 2019-2029. The broader strategy set out three strategic health pillars [3]:

1. Application of data science methods (including artificial intelligence [AI]) to diagnostic images, genomics, mobility, environmental, and other data analytical methods for automated disease diagnostics, predicting disease outbreaks, disease prevention, and identifying high-risk groups for planning and resource allocation
2. Use of AI to support junior-level and expert-level health care practitioners to make better health care decisions related to treatments and referrals in quicker time and for more people
3. Use of an integrated community and technology approach to significantly reduce maternal and child mortality.

As seen from the strategic health pillars, big data and AI are fundamental to the success of the national visions. As noted by Andreu-Perez et al [4], the phrase “Big data” is becoming a buzzword whose usage continues to double every year. They went on to outline the six Vs of big data in health care: value, volume, velocity, variety, veracity, and variability. Health care data’s clinical and public health *value* in financial terms and in health outcomes continue to drive interests in health care data globally. For instance, the total UK Health and Social Care expenditure in 2020 was 140 billion pounds representing 4% of total expenditure [5]. Similarly, the per capita national health expenditure in the United States stood at US $10,739 as of 2017 [6]. Low- and middle-income countries such as Sierra Leone continue to underperform their high-income counterparts in terms of health outcomes. This is particularly evident in the maternal and child health outcomes [7].

The *volume* of health care data generated in the United States alone in 2011 was 150 exabytes (10^18^ gigabytes) [8]. While we do not currently have such statistics for Sierra Leone, this helps to indicate the volume of data expected in a truly big data–enabled health system. One can expect individualized health care data such as vital signs, historical information, or high-resolution sound and imaging data to be the bedrock of a big data–enabled health system. The different types of data coming from different sources such as sensors, mobile apps, and hospital clinic information systems all represent the *variety* component of a big data system. Genetic, laboratory, and population health data all introduce their different challenges and perspectives to health care data. There is now increasing demand for real-time health care data: velocity. Health systems strive to improve data quality as the data travel from source to where they are used: Veracity. The quality of data is dependent on data sources, and the quality reduces with each human interface. Will a given datum be available over time? What is the guarantee of its consistency? These and other questions are addressed in the variability and trustworthiness of health care data. In Sierra Leone, the government has improved health care data reporting completeness and timeliness of reporting from the health facilities in the country.

**Figure 1:**
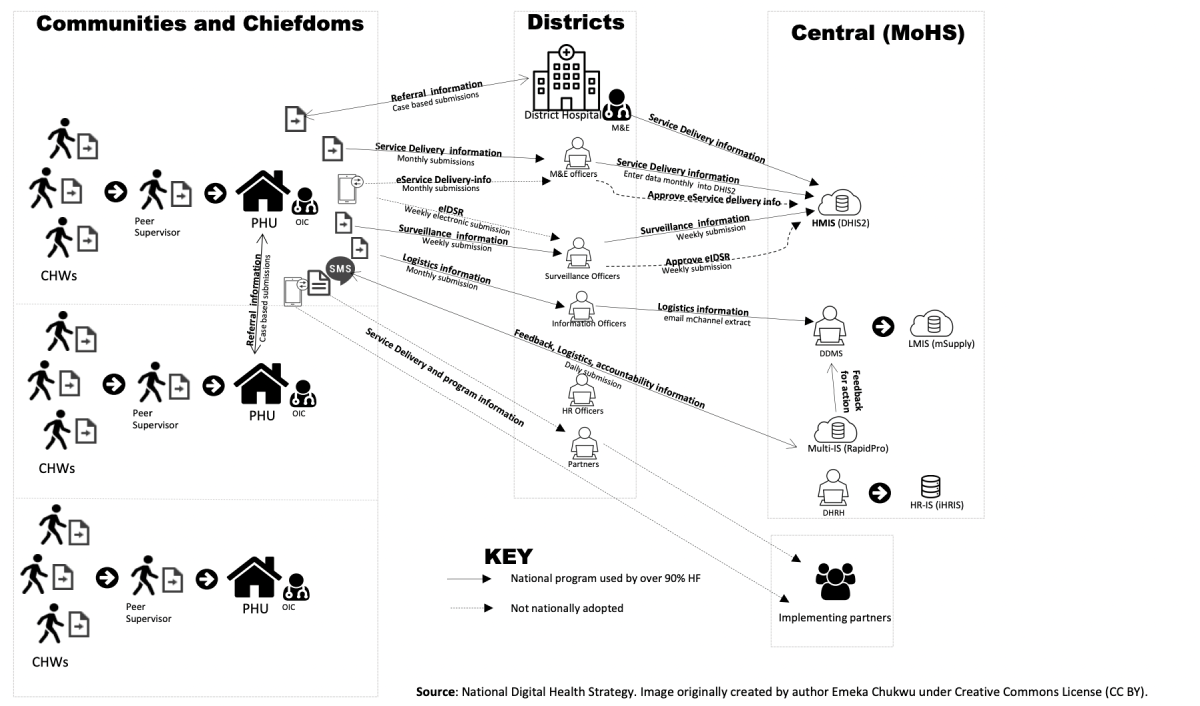
Health Information flow architecture. CHC: community health Center; CHW: community health worker; DDMS: DDMS - Directorate of Drugs and Medical Supplies; DHIS2: District Health Information Software; DHRH: District Human Resource for Health; eIDSR: electronic Integrated Disease Surveillance and Response; HF: Health Facility; HMIS: Health Management Information System; HR: human resources; HR-IS (iHRIS): Human Resources Information System; LMIS: Logistics Management Information System; M&E: Monitoring and Evaluation; MCHP: maternal and child health posts; MoHS: Ministry of Health and Sanitation; OIC: Officer in Charge; PHU: Primary Healthcare Unit.

There are 1284 health facilities in Sierra Leone, including 24 district hospitals, and the rest are primary health care units (PHUs) [9]. Each health facility service delivery point and disease surveillance unit reports through the District Health Information System (DHIS) to the MoHS at the central level [9,10]. Data flow through the different levels of information is as shown in Figure 1 from the national digital health strategy [2]. The health facility’s aggregate service delivery report submission rate was 98.6%, with 91.4% submitting on time [2].

### Study Objective

This study was commissioned to understand the different health facilities and other health systems’ digital health solutions. We also analyzed and present how these solutions are used to share information among different stakeholders in the health system, the structure of data shared, and how the data are used for decision-making. Findings from this mapping exercise conducted in 2019 provide evidence of the linkage between the availability of individualized (or longitudinal) digital health data and the exchange of these data in support of the national digital health vision. The methodology section discusses the overall investigation methodologies, including sampling, data collection, analysis, and interpretation. Next, we present our findings and the implications with key recommendations.

## Methods

### Survey Tools

For this survey, the 13 district health medical officers (DMOs) were targeted for survey. The DMOs are the health care policy implementers in their respective district and oversee district health programs at health facilities in their district. In addition, a stratified sample of 72 health facilities were also selected and visited. A separate tool was developed for digital health implementing organizations in the country. The 3 survey tools used for the survey are those shown in Table 1. Each of the questionnaires were coded into CommCare electronic mobile form [11]. Each DMO was visited, and the questionnaire was applied. Similarly, the digital health implementing organizations were visited, and a questionnaire was applied.

**Table 1:** Survey tools used for digital health mapping.

Tool name	Target respondent	Alternative	Where it is applied
Assessment Survey for District Health Management Team	District health medical officer	Authorized representative	District level
Assessment Survey for Implementing Partners	Implementing partners or implementing ministries, departments and agencies leads	Authorized representative	National or district level
Health Facility Checklist	Hospital superintendent or PHU^a^ officer in charge	Representative	Health facility

^a^PHU: primary health care unit.

### Health Facility Sampling

For the health facilities, a stratified sampling technique that includes 72 health facilities (out of 1284) with a confidence level of 95% and 11% margin of error to ensure findings can be generalizable. Health facilities were stratified into urban and rural and into high, medium, or low digital health activity health facilities, using information from the Directorate of Policy, Planning and Information (DPPI) at the MoHS working with respective DMOs. The health facilities surveyed include 17 urban and 55 rural health facilities, as shown in Table 2. In total, 96% (n=69) are public sector health facilities. The district’s DMO determines urban-rural classification. A facility is classified as low digital health activity if no digital health solution is in use at the health facility, medium if 1 or 2 solutions are used, and high if 3 or more.

These classifications were in addition to their Hospital versus PHU categorizations. In order to arrive at our sample size, a minimum of 5 health facilities were purposefully targeted for selection in each district visited. Each district DMO suggested one district hospital as part of the 5 survey health facilities. One health facility with high digital health activity was prioritized, followed by one with medium activity, followed by low (or no) activity. The process outlined above is repeated until the required number of health facilities is reached.

**Table 2:** Distribution of health facilities surveyed, by district (health facility survey).

Health facilities by district, n																																
Bo		Bombali		Bonthe		Kailahun			Kambia		Kenema			Koinadugu		Kono			Moyamba			Port loko		Pujehun			Tonkolili			Western Rural		Western Urban
R^a^	U^b^	R	U	R	U	R	U	R		U		R	U		R		U	R		U	R		U		R	U		R	U		R	
4	2	4	1	4	2	4	1	5		3		1	5		4		1	5		1	5		4		1	4		1	4		6	

^a^R: rural.

^b^U: urban.

### DMO and Implementer Sampling

In addition to health facilities, all the DMOs and all identified digital health implementers were surveyed. Implementing partner organizations were included for the structured survey if they have an active digital health implementation at the national or district level, as determined by the DPPI at the MoHS. The implementer survey tool covered the state of their digital health solutions. In total, 15 implementing organizations reported supporting digital health solutions in Sierra Leone and were all surveyed. Each implementer had one or more digital health solutions at various degrees of implementation. Similarly, the DMO—heading the District Health Management Team (DHMT)—was surveyed for the state of digital health solutions at the health facility they oversee.

### Data Collection and Analysis

Study personnel surveyed targeted respondents at the national level and then moved to the district and health facility levels. No identifiable information was collected as authorized institutional representatives were surveyed. The quantitative data collection and structured interviews were carried out using the CommCare mobile app, which facilitated automatic data transmission to the cloud for easy access. Enumerators collected data using mobile forms, which were aggregated into a Microsoft Excel spreadsheet. The aggregated data were later analyzed with “pandas” and “matplotlib” libraries of Python.

## Results

Here we present our study findings concerning the state of digital health solutions in Sierra Leone. The digital health solutions group the findings, data sharing practices, and current data use.

### Solutions (Services and Applications)

#### DHMT Survey Findings

The number and distribution from the survey is presented as reported by the DHMT and by health facilities. Based on the survey of DHMTs, Kailahun, Kenema, Karane, Pujehun, Moyamba, Freetown-Western-Rural, and Freetown-Western-Urban reported having 4 or more digital health services and applications. Bo and Kono districts had 3, and the remaining districts had 2 or fewer. Among the digital health solutions, every district used the national District Health Information Software (DHIS2).

#### Health Facility Survey Findings

**Figure 2:**
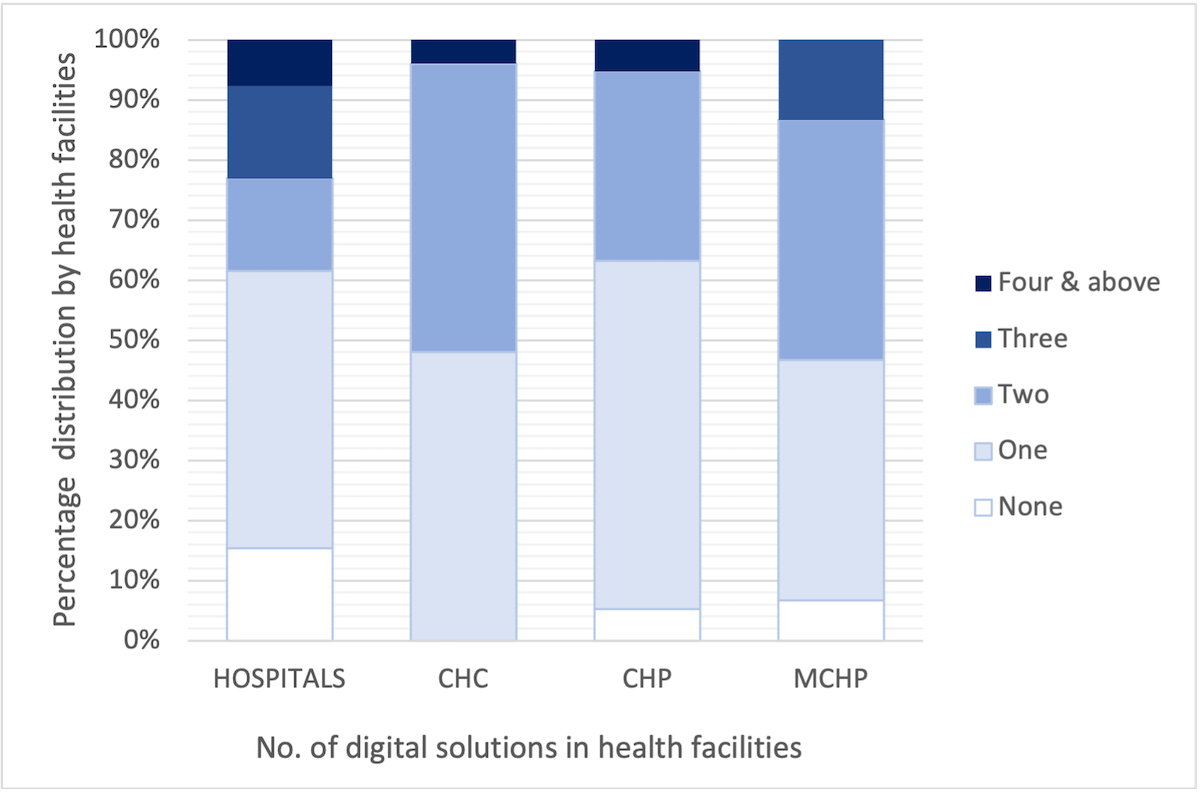
Health facilities by the number of digital health activities (health facility survey). CHC: community health centerl; CHP: community health post; MCHP: maternal and child health post; No: number.

Similarly, the health facility survey showed that in total, 3 health facilities had 4 or more digital health services and applications in use, 4 facilities had 3 solutions in use, and the majority had 2 services and applications in use or only the DHIS for aggregate reporting. Figure 2 provides a breakdown of this distribution by hospitals and the different PHUs. Based on health facility respondents, digital tools used by health facilities were aggregate electronic Integrated Disease Surveillance (eIDSR) and DHIS2. Others include the case-based reporting tool based on odk, commcare, ihris, vaxtrac, and healthConnect. Some health facilities also indicated using SMS reporting through RapidPro and the NHMIS-paper-form HF1_HF9 reporting tool.

Almost all facilities reported that the services and applications were functional, except a negligible few, which were reported not to be working at the time of the data collector’s visit. The World Health Organization (WHO) classified digital health interventions into client, health care provider, health system administrator, and data services–facing services and applications (solutions) [12]. Based on these categorizations, the majority of the services and applications deployed were either for data services or for health care providers. Table 2 shows the distribution and purpose of these digital health solutions by health facility type. SA_1 represents the first service and application, and SA_5 represents the fifth service and application. The first column in Table 3 implies that 5 hospitals had at least 1 service and application that are used for data service. In addition, there is one hospital whose fourth service and application are used for data services, and one hospital whose fifth service and application are used for data services.

**Figure 3:**
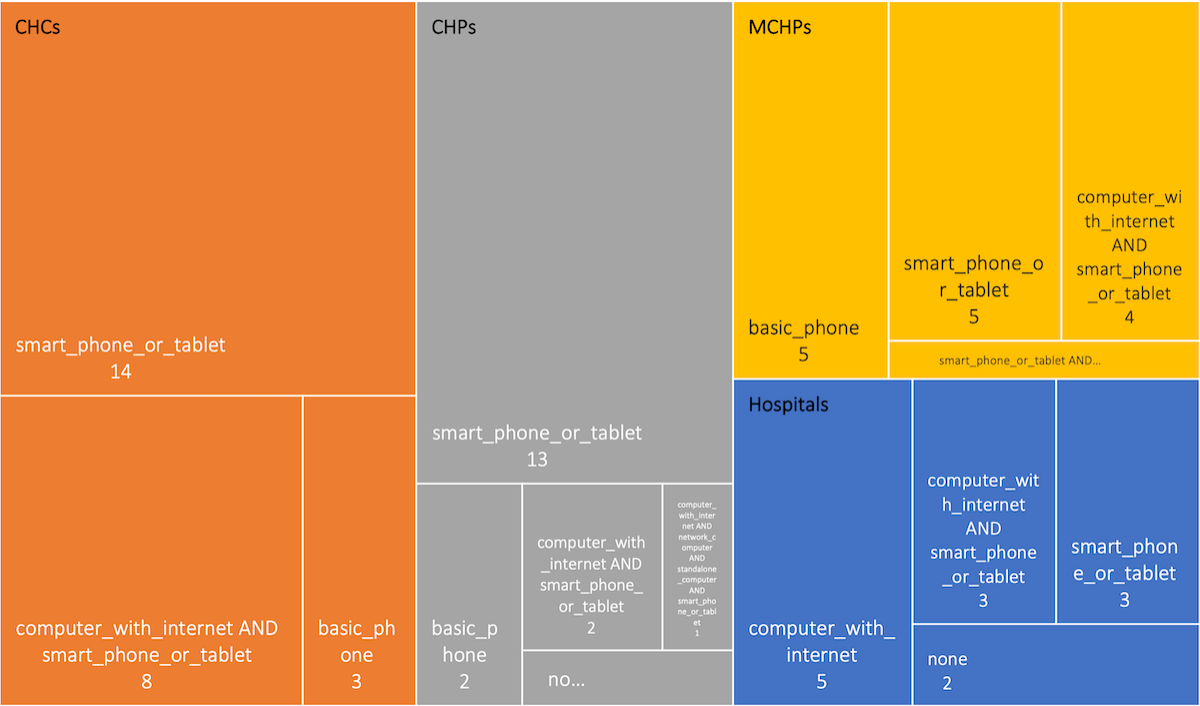
Access techniques used for digital health services and applications (health facility survey). CHC: community health center; CHP: community health post; MCHP: maternal and child health post.

Respondents at the health facilities surveyed were asked about how the services and applications were accessed at their facilities. The hospitals accessed their digital health solutions mainly using computers and through the internet. Similarly, the PHUs accessed their digital health solutions primarily using tablets (or smartphones) (Figure 3), although the maternal and child health posts used more basic phones than the other PHUs on average. Each rectangle represents access type, relative sizes of each rectangle indicate the number of health facilities, and the color indicates the health facility type.

**Table 3:** Purpose of the digital health services and applications (health facility survey).

Health facility type and purpose of the services and applications			Number of health facilities								
			SA_1		SA_2		SA_3		SA_4		SA_5
**Hospital**											
	data_services	5		0		0		1		1	
	healthcare_provider data_services	4		3		0		0		0	
	client data_services	1		1		0		0		0	
	client healthcare_provider, health_systems_administrator, and data_services	1		0		0		0		0	
	health_systems_administrator	1		0		0		0		0	
	healthcare_provider, health_systems_administrator, and data_services	1		1		0		0		0	
	health_systems_administrator and data_services	0		0		2		0		0	
	Client	0		0		1		0		0	
**Community health centers**											
	healthcare_provider and data_services	8		1		0		0		0	
	healthcare_provider, health_systems_administrator, and data_services	8		4		0		0		0	
	data_services	4		5		0		0		0	
	Client and data_services	2		0		0		0		0	
	Client, healthcare_provider, health_systems_administrator, and data_services	1		2		1		0		0	
	health_systems_administrator and data_services	1		1		0		0		0	
	healthcare_provider	1		0		0		0		0	
	Client and healthcare_provider	0		0		0		1		0	
**Community health posts**											
	healthcare_provider and data_services	10		2		0		1		0	
	data_services	2		0		0		0		0	
	Client	1		1		0		0		0	
	Client and data_services	1		1		0		0		0	
	Client, healthcare_provider, and data_services	1		1		0		0		0	
	Client, healthcare_provider, health_systems_administrator, and data_services	1		0		1		0		0	
	Client and health_systems_administrator	1		0		0		0		0	
	health_systems_administrator	1		0		0		0		0	
	health_systems_administrator and data_services	1		1		0		0		0	
	healthcare_provider	1		0		0		0		0	
**Maternal and child health posts**											
	data_services	4		2		1		0		0	
	healthcare_provider and data_services	4		0		0		0		0	
	Client and data_services	2		1		0		0		0	
	healthcare_provider, health_systems_administrator, and data_services	2		2		0		0		0	
	health_systems_administrator	1		1		1		0		0	
	health_systems_administrator and data_services	1		1		0		0		0	
	healthcare_provider and health_systems_administrator	1		0		0		0		0	
	Client, healthcare_provider, health_systems_administrator, and data_services	1		0		0		0		0	

#### Implementing Partner Survey Finding

Implementing partners used the services and applications shown in the word-art in Multimedia Appendix 1. The majority of the tools used were for data collection, processing, and reporting. The majority of the implementing partners supported the use of the DHIS, either through the national instance or a different instance. All surveyed implementing organizations noted that the status of their digital health effort was “active and working.”

### Information Sharing

The findings from all the 13 districts show that all DHMTs share both service delivery and implementation information with health facilities in their district. All but one share (or send) both implementation and service delivery data with the central MoHS, nongovernment organizations (NGOs) or other implementing organizations. In total, 6 districts shared this information via email, 6 shared it in print format, and 3 reported sharing data via SMS. All districts submit data to the NHMIS web portal in the required format every month. Health facility information sharing is along the aggregate data reporting only to the DHMT or supporting NGOs. At PHUs, 78% share aggregate data with the DHMT only, and the other 22% share the aggregate data with the DHMT and NGOs. Similarly, hospitals share aggregated service delivery and implementation data with 16% either sharing to NGO or to no one as shown in Table 4. No health facility or district shares individualized information between different applications.

The majority of the implementing partners reported having a written standard operating procedure to facilitate data exchange at the health facilities they supported. Almost all partners surveyed shared data in a government-approved format, in addition to other formats. The majority of the partners shared data with health facilities, DHMTs, and the MoHS.

**Table 4:** Distribution of where aggregate data are sent by hospitals (health facility survey).

Where the data are sent	Proportion, %
To no one	8
District Health Management Team	23
Central Directorate of Policy, Planning and Information	23
District Health Management Team and central Directorate of Policy, Planning and Information	23
District Health Management System, nongovernment organizations, and central Directorate of Policy, Planning and Information	15
Nongovernment organizations	8

### Data Use

All surveyed health facilities reported using data for decision-making.

## Discussion

### Solutions (Services and Application)

**Figure 4:**
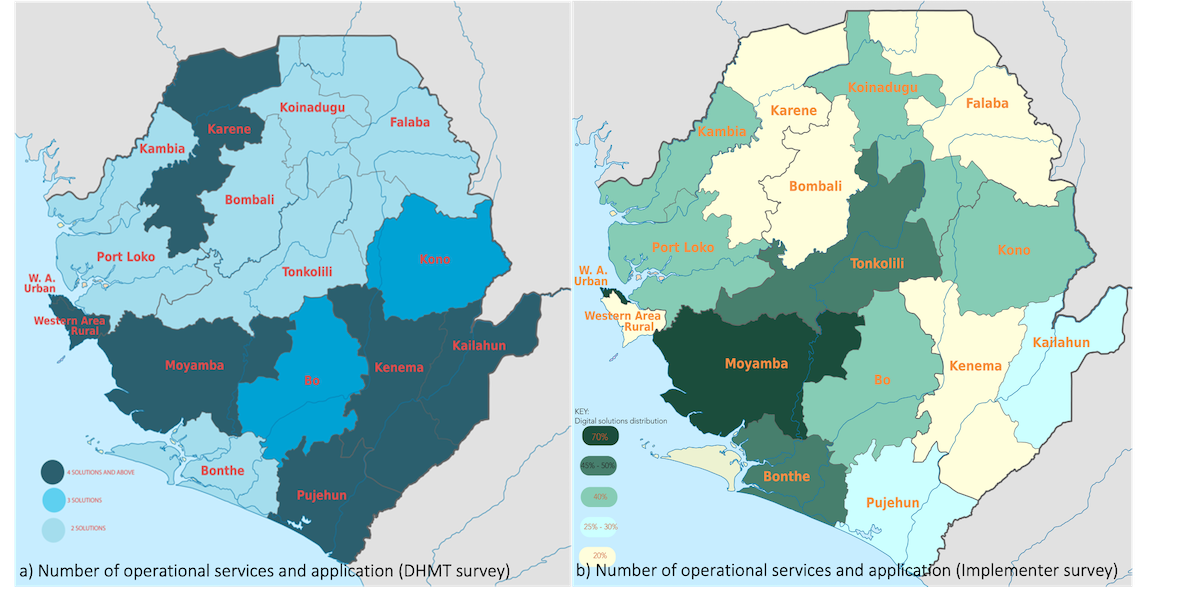
Number of operational services and application per district. (a) Number of operational services and applications (District Health Management Team survey); (b) number of operational services and applications (implementer survey).

Using the three structured interview approaches to determine the status of digital health solutions deployed in Sierra Leone highlighted the different dimensions and the distribution of these solutions. As shown in Figure 4a, the DHMT survey shows an even distribution of these tools across the districts. However, the implementing partner survey shows that the distribution is skewed to 2 districts, as shown in Figure 4b. Another dimension of this survey from the health facilities shows that most PHUs have 2 or more digital health services and applications. Furthermore, the majority of these solutions are intended for data services and use tablets as access mechanisms. On the other hand, hospital solutions use computers alone or computers and tablets. This is expected as the workload at hospitals often requires bigger-form factor hardware devices. Furthermore, hospitals have better electricity and internet-network infrastructure to support using computer-based services and applications (as against tablet-based services).

### Interoperability

Although stakeholders share information within and across institutions in government data sets, the structure and format of these data vary greatly (email, SMS, paper forms, and portal reporting). The implication of our findings is that no digital health foundational registry (patient, provider, practitioner, and health terminology classification) is used by any of these tools. There should be coordination around a standardized data format to reduce duplication among implementing partners, especially for individualized data-based solutions. Data-intensive digital health solutions should improve the feedback loop and data use, especially at health facility levels [13]. License-free individualized data sharing standards such as the Fast Healthcare Interoperability Resource should be explored [14]. The WHO’s International Classification of Diseases or Systematize Nomenclature of Medicine Clinical Terms will be invaluable in designing an interoperability terminology interface as proposed in the digital health strategy [15]. In order to mitigate data blocking as classified by the US Health Insurance Portability and Accountability Act (HIPAA) [16], blockchain, an emerging technology that allows shared ownership and administration of data, can be used [17]. Data blocking has been responsible for limited interoperability, and low- and middle-income countries often use western regulations such as the HIPAA as the best practices benchmark. An insufficient feedback loop was identified by the surveys, especially at health facilities.

### Big Data Opportunities and Digital Health Vision

Application of big data in health care can be critical, and evidence from around the world supports this [18]. However, different surveys of the application of big data in health care show that these applications use individual-level data rather than aggregate-level information [18]. Given that no solution currently shares or exchanges individual-level data (longitudinal patient data), opportunities for using facility-generated big data at present are greatly limited to aggregate disease surveillance only. Steps necessary to improve individualized information sharing are critical to achieving the vision of digital health. This can also impact the quality of health system data and the ability to use the data. Investment in a multi-sourced data triangulation system will be a low-hanging fruit for data interoperability and use especially at district levels.

### Limitations

Given that the digital ecosystem is evolving, and owing to the rapid deployment of digital interventions fueled by the pandemic, we acknowledge that new solutions may have been deployed after the survey. However, this snapshot mapping will prove invaluable to policy makers. In addition, other key enabling environment components such as digital health infrastructure, workforce capacity, and funding remain key barriers to achieving these ideals.

### Conclusions

This mapping from frontline health workers, policy makers, and implementers has shown that there are many digital health solutions in operation at health facilities in Sierra Leone. This study also shows that only aggregate-level data are shared for reporting and monitoring purposes only. Individualized information (or longitudinal patient data) is not currently processed for exchange among different solution providers. Hospitals mostly use computer-based solutions, while PHUs mostly use tablet-based solutions. No foundational digital health registry is used by any of the surveyed and mapped digital health solutions.

There are opportunities to leverage the 6 Vs of big data (value, volume, velocity, veracity, variability, and variety) to achieve the national digital health vision. Integrated care resulting from big data–facilitated electronic health records is only possible through individualized data-enabled care coordination [19].

## Appendix

Multimedia Appendix 1 Names of digital health services and applications (implementers’ survey).

## Abbreviations

AI: artificial intelligence
DHIS2: District Health Information Software
DHMS: District Health Management System
DHMT: District Health Management Team
DMO: district health medical officer
DPPI: Directorate of Policy, Planning and Information
eIDSR: electronic Integrated Disease Surveillance and Response
HIS: health information system
MoHS: Ministry of Health and Sanitation
NGO: nongovernment organization
NHMIS: National Health Management Information System
PHU: primary health care units
