# Supplementary figures and images for: Digital Health Solutions and State of Interoperability: Landscape Analysis of Sierra Leone
Source: JMIR Form Res. 2022 Jun 10;6(6):e29930. doi: 10.2196/29930 (PMC9233249; doi:10.2196/29930)

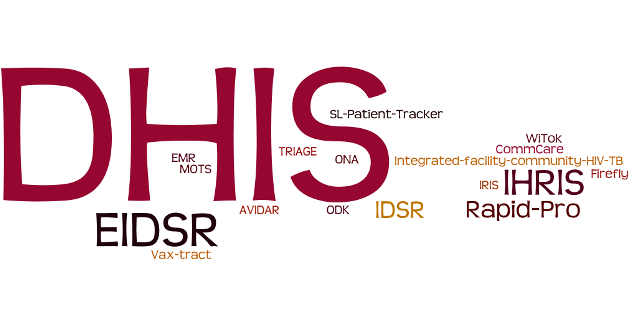

Supplement: Multimedia Appendix 1 [file formative_v6i6e29930_app1.png]
